# Redistributive effects of China's urban–rural resident basic medical insurance: a theoretical model and empirical analysis

**DOI:** 10.3389/fpubh.2025.1689510

**Published:** 2025-11-05

**Authors:** Wenfang Ji, Fuling Chu, Yi Qin

**Affiliations:** School of Insurance, Central University of Finance and Economics, Beijing, China

**Keywords:** healthcare reimbursement, urban-rural resident basic medical insurance, income redistribution, Gini coefficient, MT index

## Abstract

**Introduction:**

As a core pillar of China's social-security system, the Urban–Rural Resident Basic Medical Insurance (URRBMI) redistributes income and promotes equitable access to health care, offering globally relevant lessons for similar economies. Despite its rapid expansion, the redistributive performance of URRBMI has not been rigorously assessed. Clarifying the conditions under which it narrows or widens income gaps—and the extent to which it does so—is therefore essential for evidence-based policy reform.

**Methods:**

We develop an institutional-level theoretical model that treats the Gini coefficient of disposable income as the primary redistributive indicator. Using household-level data, we compute Gini coefficients before and after insurance reimbursement; a post-reimbursement decline denotes positive redistribution, whereas an increase signals negative redistribution. The analysis disaggregates medical expenditure into low, medium, and high tiers and compares outcomes across insured and uninsured groups. Region-specific estimates are produced for Northeast, Central, and other macro-regions to capture spatial heterogeneity.

**Results:**

Empirical estimates for the national sample confirm that URRBMI reduces income inequality overall. Redistribution is strongest in the Northeast and weakest in Central China. Expenditure-level analysis shows that when inpatient expenses lie below the deductible, the scheme exerts no redistributive force; once the deductible is crossed, reimbursements narrow income disparities, and the magnitude of redistribution rises with the reimbursement rate. Relative to the uninsured, non-participation is advantageous only when inpatient spending remains below the deductible; beyond the deductible, participation and reimbursement yields superior and progressively stronger redistributive outcomes as expenditure tiers increase.

**Discussion:**

URRBMI still faces three key challenges: the contribution mechanism is not income-related, benefit packages vary markedly, and the fund relies heavily on fiscal transfers while exhibiting limited risk-pooling capacity. To enhance redistributive effectiveness, we recommend mandatory enrolment with income-proportional contributions, higher benefit levels, and raising the pooling level of the basic medical insurance fund, while merging the urban employee basic medical insurance scheme with the urban and rural residents' basic medical insurance scheme.

## 1 Introduction

Globally, catastrophic out-of-pocket (OOP) health spending erodes households' capacity to purchase essential goods such as food, clothing or shelter, constitutes a principal external driver of financial collapse and undermines equity in health welfare. Data show that in 2022 the European Union (EU) health expenditure accounted for 8.1% of GDP (1), with about 15% funded through out-of-pocket payments (2). In parts of Eastern Europe and Central Asia the OOP share exceeds 50% and in some cases 70% (3), placing a heavy financial burden on patients and their families. According to the 2023 joint report by the World Health Organization and the International Bank for Reconstruction and Development/World Bank, the number of individuals facing catastrophic health payments—defined as OOP spending exceeding 10 percent of household budgets—rose steadily from 588 million in 2000 to 937 million in 2015 and reached 1.04 billion in 2019, implying an average annual increase of roughly 24 million people over 2000–2019. Concurrently, 344 million persons were pushed into or further into extreme poverty by OOP medical expenses in 2019, accounting for almost half (41%) of the global extreme poor that year (4).

Basic medical insurance averts catastrophic out-of-pocket spending by expanding access to essential care, thereby reducing poverty and health inequality (5). Across Europe, policymakers continue to dismantle barriers and promote equitable access to health services (6). In April 2023, Croatia amended its Health Care Act to restructure the delivery system, narrowing health inequalities by improving the geographical and financial accessibility of services (7). Finland carried out the largest reorganization of its health and social-care system to date in 2023, creating 21 wellbeing services counties that pool risks and funds nationwide (8). The reform strengthens the financial basis of service provision, guarantees equal access to health and social services, and effectively reduces inequalities in health and wellbeing (9). Egypt, Morocco, Zambia, Kenya, and Tunisia have extended statutory coverage to informal, temporary, and rural workers through new legislation; Zambia's National Health Insurance Act No. 2 of 2018, for example, introduced a compulsory scheme (10). Rwanda's community-based health insurance now covers most of the rural and informal population, achieving the highest coverage rate in sub-Saharan Africa and a positive distributional impact, and was awarded ISSA's 2019 Outstanding Social Security Achievement Award (11). Yet the redistributive yield remains modest. Flat-rate contributions are largely decoupled from individual health risk (12) and—except in Denmark (1.1%), Sweden (1.8%), and Norway (4.2%)—basic health insurance reduces income inequality by less than one percentage point in all countries surveyed (13), thereby yielding only marginal narrowing of pre-existing social-welfare gaps. Designing a scheme that both effectively buffers health shocks and narrows income disparities has therefore become a common policy imperative for advanced and developing economies alike.

China's Urban–Rural Resident Basic Medical Insurance (URRBMI) offers a common policy mirror for advanced and developing economies alike: its innovations, gains, and remaining gaps in expanding coverage, curbing catastrophic spending, and narrowing health inequities yield ready-to-use lessons and reform blueprints. As a cornerstone of China's social-security system, URRBMI narrows national income gaps through health coverage and redistribution. By the end of 2023, URRBMI covered 962.944 million individuals −72.19% of all participants in China's basic medical-insurance system—making it a cornerstone for expanding basic coverage and ensuring access to care for the sick. Between 2018 and 2023, the number of URRBMI enrollees declined by 6.31%, from 1,027.778 million to 962.944 million, primarily because some insured persons migrated to the Urban Employee Basic Medical Insurance (UEBMI) while others dropped out altogether. Despite this contraction, the scheme's fund scale and benefit generosity have improved markedly. Nominal URRBMI revenues rose 34.71%, from RMB 784.64 billion in 2018 to RMB 1,056.97 billion in 2023; expenditures increased 46.96%, from RMB 711.59 billion to RMB 1,045.77 billion; and cumulative reserves expanded 63.40%, from RMB 469.01 billion to RMB 766.37 billion (14). Concurrently, the number of reimbursed service utilizations climbed 61.11%, from 1.62 billion in 2018 to 2.61 billion in 2023. In 2023 alone, insured residents incurred RMB 1,958.156 billion in medical expenses, up 19.4% year on year, while the in-directory inpatient reimbursement ratio reached 68.1% (15).

Yet the challenges facing URRBMI should not be overlooked. Owing to the fragmented architecture of China's basic medical-insurance system, URRBMI lags far behind the Urban Employees' Basic Medical Insurance (UEBMI) in both design and operation. In 2023, URRBMI covered 72.19% of all participants in the basic medical-insurance schemes, but its revenue accounted for only 31.55% of the national basic-insurance total, its expenditures for 37.07%, and its accumulated surplus for merely 15.98%. While URRBMI has been the main driver of expanding population coverage, its comparatively shallow financing base inevitably yields markedly lower benefit generosity than UEBMI. The flat-premium mechanism—largely decoupled from household income—has depressed participation, while wide inter-regional benefit disparities and shallow pooling of risk at low administrative tiers have further eroded both the protective capacity and the redistributive bite of URRBMI.

Most studies deem basic medical insurance progressive (16–21), but emerging work—owing to different estimators and assumptions—argues it may widen income dispersion (22, 23). Zhou et al. (24) find that the New Rural Cooperative Medical Scheme (NRCMS) exerts a weak redistributive effect in the short run and becomes disequalising over time. Medical spending itself is regressive, and current reimbursement rules are too shallow to neutralize this bias (25), so the scheme can amplify rather than attenuate inequality (26). The literature suggests that two factors drive the conflicting results. First, programme designs differ: NRCMS, URRBMI, and UEBMI rely on markedly distinct financing and benefit formulas, yielding heterogeneous distributive outcomes. Second, the treatment of out-of-pocket payments is decisive. When all OOP costs are treated as the end-product of insurance, estimates tend to show regressivity; once spending is properly disentangled, the system usually appears progressive. Redistribution through health insurance unfolds in four sequential stages: pre-enrolment, post-contribution, incidence of medical spending, and final reimbursement. The redistributive bite occurs neither at the contribution stage nor through the volume of spending *per se*, but in the reimbursement episode that ultimately determines net household income.

Against this backdrop, we compare inequality after reimbursement with inequality observed immediately after out-of-pocket payments but before reimbursement, thereby isolating the net redistributive effect of URRBMI. Tracing the complete institutional chain—from contribution design to benefit payment—we unpack its redistributive logic and construct an empirically testable theoretical model. Using the nationally representative 2019 China Household Finance Survey (CHFS), we quantify redistribution at the national, regional, and low-/medium-/high-expenditure tiers, evaluate scheme performance, identify design flaws, and propose targeted improvements. Our study offers two contributions: (i) theoretical: modeling results show that URRBMI's redistributive power may be limited in specific cases but is significantly inequality-reducing overall, enriching the social health-insurance literature and correcting the prevailing view that basic medical insurance is regressive. (ii) Practical: leveraging CHFS micro-data, we provide the first systematic, multi-dimensional yardstick of URRBMI's redistributive impact; grounded in operational realities, we deliver an actionable reform package covering financing rules, benefit design, and fund pooling that both strengthens URRBMI's redistributive capacity and serves as a ready reference for other countries grappling with similar health-care reform dilemmas.

## 2 Institutional analysis

In 2016, China's State Council issued the “Guiding Opinions on Integrating the Basic Medical Insurance Systems for Urban and Rural Residents” (Guofa [2016] No. 3), formally merging the former Urban Resident Basic Medical Insurance and the New Rural Cooperative Medical Scheme into the unified Urban-Rural Resident Basic Medical Insurance (hereafter URRBMI). The redistributive effect of URRBMI is realized by collecting flat-rate premiums and then providing benefit payments to insured individuals who incur medical expenses, thereby achieving secondary redistribution across income groups. The effectiveness of this function hinges on two key design features.

1. Contribution mechanism. URRBMI employs an annual, per-capita flat premium. Individuals are the primary contributors, while governments provide targeted subsidies. The 2007 State Council Guidelines on Piloting the Urban Resident Medical Insurance stipulated that only a social-pooling fund would be established—no individual medical savings accounts would be created. At inception, the central government provided a minimum annual subsidy of RMB 40 per enrollee. An additional RMB 10 per student or child from low-income households or with severe disabilities was granted, and at least RMB 60 per year was added for other vulnerable groups, such as low-income seniors over 60 or severely disabled non-working adults (27). Pursuant to State Council Document Guofa [2016] No. 3, collective or other socio-economic organizations are also encouraged to supplement contributions. In August 2024, the National Healthcare Security Administration (NHSA), together with the Ministry of Finance and the State Taxation Administration, raised the 2024 individual premium by RMB 20, setting the new floor at RMB 400 per person per year (28).
2. Benefit design. As stipulated in the 2003 Circular of the General Office of the State Council on Forwarding the Opinions of the Ministry of Health and Other Departments on Establishing the New Rural Cooperative Medical Scheme, the NRCMS fund shall primarily subsidize participating farmers' expenditures on major medical events or inpatient care. The 2009 joint guidance on consolidating and developing NRCMS lifted the cap to at least six times local per-capita net rural income (29). The 2009–2011 near-term medical and healthcare system reform planextended the same principle to urban residents, raising the ceiling to roughly six times local per-capita disposable income (30). The 2010 Ministry of Human Resources and Social Security notice set the strategic target of reimbursing 60% of in-policy inpatient costs nationally, and 70% at secondary-level or lower medical facilities (31).

## 3 Theoretical model

Building on the institutional analysis above, China's Urban–Rural Resident Basic Medical Insurance (URRBMI) relies on a flat-rate premium and reimburses two broad categories of care: inpatient services and non-inpatient services (the latter including outpatient pooling benefits). The rules governing inpatient reimbursement are characterized by three parameters: the deductible, the ceiling, and the coinsurance rate. Specifically, deductible: the amount an enrollee must pay out-of-pocket before the insurer begins to reimburse. Coinsurance rate: the proportion of eligible expenses reimbursed between the deductible and the ceiling. Ceiling: the maximum amount the reimbursing agency will cover for a single reimbursement claim. Because provincial policies differ, these three parameters vary widely across regions. In general, the higher the hospital tier, the lower the reimbursement rate. Taking Beijing's 2024 URRBMI schedule as an example: for inpatient care at primary-level hospitals and below, the deductible is RMB 300 and the reimbursement rate is 80%. For inpatient care at secondary-level hospitals, the deductible is RMB 800 and the reimbursement rate is 78%. For inpatient care at tertiary hospitals, the deductible is RMB 1,300 and the reimbursement rate ranges from 75 to 78%. The reimbursement ceiling is RMB 250,000 (32). Because inpatient episodes account for the bulk of medical spending, they provide a clear signal of the redistributive impact of the benefit package. The theoretical model below therefore adopts inpatient reimbursement as the illustrative case and calibrates its key parameters accordingly.

### 3.1 Redistributive theoretical indicators

#### 3.1.1 Theoretical indicators

According to the 2023 Statistical Communiqué, China's annual per-capita disposable income is RMB 39,218 (33); we round this to RMB 40,000 and denote it as *W*_*r*_. To simplify the exposition, the population is split into five equally-sized income quintiles whose pre-tax incomes are set at 0.3*W*_*r*_, 0.6*W*_*r*_, 1.0*W*_*r*_, 2.0*W*_*r*_, and 3.0*W*_*r*_ respectively. Taking Beijing's 2024 URRBMI schedule as the benchmark, we fix the following parameters: flat premium: RMB400 → 0.01*W*_*r*_. Deductible (secondary-level hospital): RMB800 → 0.02*W*_*r*_. Coinsurance rate (secondary-level hospital): 78% (uniform across quintiles). Statutory ceiling: 6*W*_*r*_.

To avoid negative incomes after deducting inpatient expenditures, we assign each quintile a modest medical-expenditure amount equal to 0.02*W*_*r*_, 0.08*W*_*r*_, 0.14*W*_*r*_, 0.20*W*_*r*_, or 0.26*W*_*r*_ respectively (the first value also captures the case of zero spending). Table 1 summarizes the key parameters of the theoretical model.

**Table 1:** Construction of key indicators in the theoretical model of URRBMI.

**Indicator**	**Empirical benchmark**	**Theoretical calibration**
Average annual disposable income of urban and rural residents	RMB 40,000	*W*_*r*_
Five income groups	0.3, 0.6, 1, 2, and 3 times the annual per-capita disposable income	0.3*W*_*r*_/0.6*W*_*r*_/*W*_*r*_/2*W*_*r*_/3*W*_*r*_
Flat-rate URRBMI premium	RMB 400	0.01*W*_*r*_
Deductible	RMB 800 (secondary-level hospital, Beijing 2024)	0.02*W*_*r*_
Reimbursement rate	78% (secondary-level hospital, Beijing 2024)	78%
Medical-expenditure brackets	The deductible is set at 0.02*W*_*r*_, followed successively by 0.08, 0.14, 0.20, and 0.26 times the local annual per-capita disposable income	0.02*W*_*r*_/0.08*W*_*r*_/0.14*W*_*r*_/0.2*W*_*r*_/0.26*W*_*r*_

Source: compiled by the authors.

Assume that the probability of incurring each level of inpatient cost differs across income groups. According to the OECD (34), low-income individuals have weaker health-seeking behavior and lower preventive-care uptake, which is concentrated among higher-income groups. Consequently, as income rises, people adopt healthier lifestyles, attend routine check-ups and treat minor illnesses early, raising the likelihood of low-cost episodes and reducing that of high-cost admissions. The opposite holds for lower-income groups, who often delay care and are more prone to high-cost hospitalisations. We therefore parameterise the quintile-specific probability distributions as follows (Table 2): for the lowest-income quintile, the probabilities of incurring low, lower-mid, mid, upper-mid and high inpatient expenditures rise linearly from 0.10 to 0.30 in 0.05 increments. For the lower-middle-income quintile, they increase from 0.15 to 0.25 under the same step. The middle-income quintile is assigned a uniform 0.20 across all cost bands. The upper-middle-income quintile sees probabilities decline from 0.25 to 0.15. The highest-income quintile follows a downward gradient from 0.30 to 0.10.

**Table 2:** Probability distribution of inpatient expenditure across cost bands by income group.

**Probability distribution**		**Income group**				
		**Low-income group 0.3W**_**u**_	**Lower-middle-income group 0.6W**_**u**_	**Middle-income group** **W**_**u**_	**Upper-middle-income group 2W**_**u**_	**High-income group 3W**_**u**_
Inpatient expenditure tiers	0.02*W*_*r*_ (low: deductible)	0.1	0.15	0.2	0.25	0.3
	0.08*W*_*r*_ (lower-mid-tier inpatient expenditure)	0.15	0.175	0.2	0.225	0.25
	0.14*W*_*r*_ (mid-tier inpatient expenditure)	0.2	0.2	0.2	0.2	0.2
	0.2*W*_*r*_ (upper-mid-tier inpatient expenditure)	0.25	0.225	0.2	0.175	0.15
	0.26*W*_*r*_ (high-tier inpatient expenditure)	0.3	0.25	0.2	0.15	0.1

#### 3.1.2 Measurement indicators

**Figure 1:**
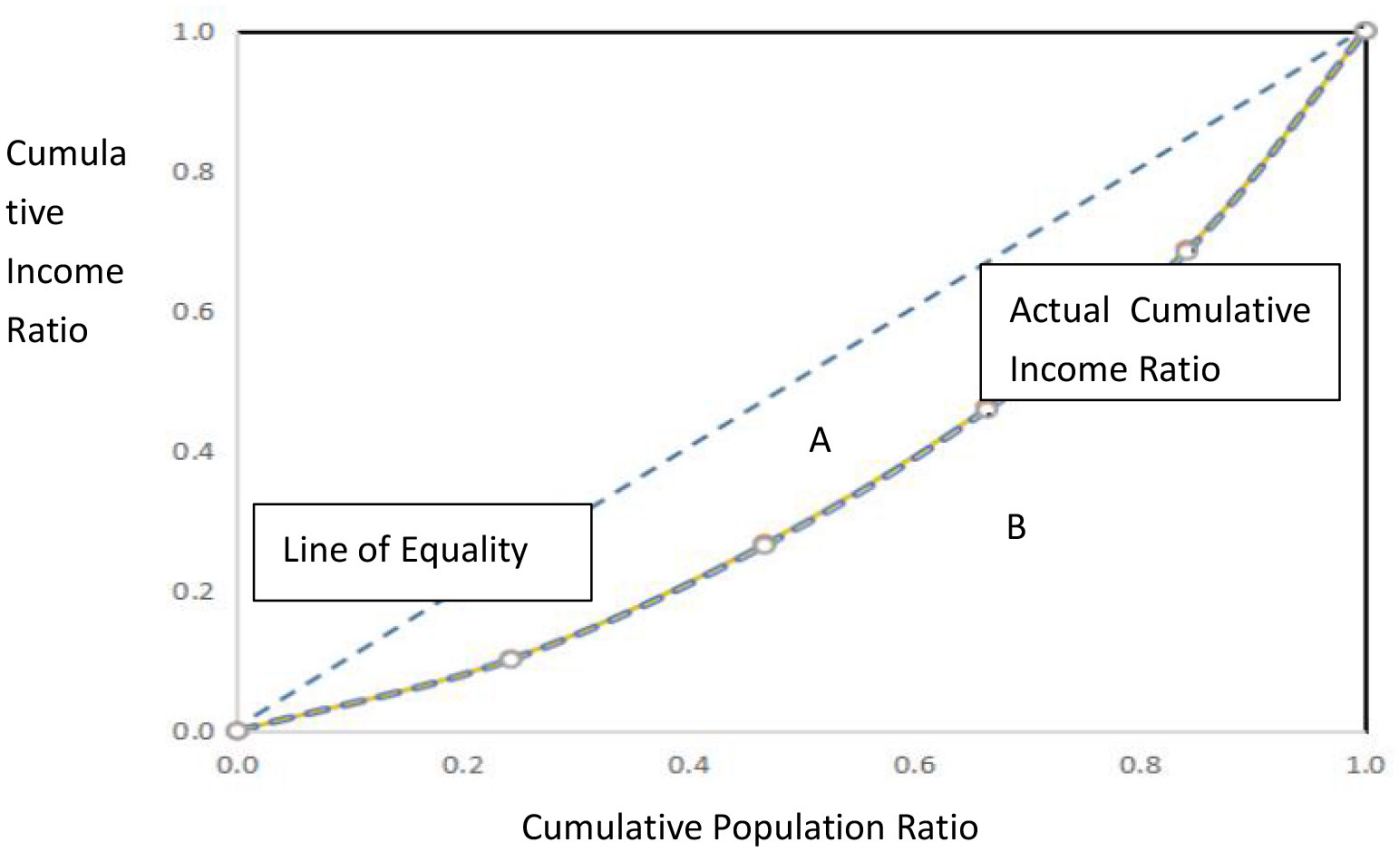
Schematic illustration of the Lorenz curve and the Gini coefficient.

The redistributive impact is assessed using three standard indices: the Gini coefficient, the Musgrave-Thin (MT) index, and the Relative Redistribution Effect (RMT) index. The schematic illustration and computational logic of the Gini coefficient are presented in Figure 1: after ranking individuals from lowest to highest income, divide the covered population into n equally sized groups. Let *p*_*t*_ be the cumulative population share and *q*_*t*_ the cumulative income share. Plotting ( *p*_*t*_, *q*_*t*_) yields the Lorenz curve. Denote the area between the Lorenz curve and the 45° line of perfect equality as A, and the area beneath the Lorenz curve as B. The Gini coefficient G = $\frac{A}{A+B}$

$B=\frac{1}{2}\overset{n-1}{\underset{t=0}{\sum }}\left(p_{t+1}-p_{t}\right)\left(q_{t+1}+q_{t}\right)$

$G=\frac{A}{A+B}=\frac{\frac{1}{2}-B}{\frac{1}{2}}=1-2B=1-2^{*}\frac{1}{2}\overset{n-1}{\underset{t=0}{\sum }}\left(p_{t+1}-p_{t}\right)\left(q_{t+1}+q_{t}\right)$

$=\overset{n-1}{\underset{t=0}{\sum }}{(p}_{t}q_{t+1\;}-p_{t+1}q_{t})$

Here, Gε[0, 1]; the larger the value of G, the greater the inequality. G = 0 signifies perfect equality, whereas G = 1 denotes complete inequality.

The Musgrave-Thin (MT) index quantifies how the income gap changes for each income group before and after the scheme's benefits (35). Specifically, MT = G - G^*^. where G = Gini coefficient after out-of-pocket medical expenses but before insurance reimbursement; G^*^ = Gini coefficient after insurance reimbursement. MT > 0 ⇒ the income gap narrows (positive redistribution); MT < 0 ⇒vthe gap widens (negative redistribution). Because the MT index measures the absolute change in inequality, it is also termed the absolute redistributive effect.

The Relative Redistributive Effect (RMT) expresses the MT index as a percentage of the pre-reimbursement Gini coefficient to measure the relative intensity of redistributive effect (36):

*RMT*_*m*_= ${\frac{G-G^{*}}{G}}^{*}100\%={\frac{MT}{G}}^{*}\;100\%.$

For URRBMI, *RMT*_*m*_ tells us how much the benefit payment compresses the pre-reimbursement income dispersion, giving policymakers an intuitive gauge of the scheme's distributional leverage.

### 3.2 Measurement and results

The theoretical model is evaluated under two mutually exclusive scenarios. Scenario 1: redistribution among the insured who actually claim benefits. Although URRBMI's fiscal flow passes through premium collection, medical expenditure and reimbursement, the decisive redistributive moment is the reimbursement episode. Assume that every income quintile faces a non-zero risk of illness and hospitalization, but the probabilities of incurring each cost-band expenditure differ across quintiles. We compare the Gini coefficient after out-of-pocket payment (G) with the Gini coefficient after the insurance benefit is paid (G^*^). The redistributive effect of the resident medical insurance scheme is measured using the MT index (absolute effect) and the *RMT*_*m*_(relative effect). Scenario 2 compares the redistributive impact of enrolling in URRBMI vs. remaining uninsured. The second exercise contrasts the income distribution of the entire insured pool (Scenario 1) with that of a counter-factual population in which no one is covered by URRBMI. The difference between the two post-expenditure Ginis yields an MT index that captures the net redistributive impact of merely participating in the scheme, ceteris paribus. The results for both scenarios are presented sequentially below.

#### 3.2.1 Scenario 1 redistribution among the insured who actually receive reimbursement

For every income quintile the redistributive path involves four sequential states:

1. Pre-enrolment. Disposable income equals the initial disposable income.
2. Premium payment. Disposable income after contributions = initial disposable income—individual premium paid.
3. Out-of-pocket payment for care. Disposable income after self-payment = disposable income after contributions—out-of-pocket medical expenditures paid in advance, weighted by the probability of incurring this cost-band inpatient episode.
4. Insurance reimbursement. Final disposable income = Disposable income after self-payment + URRBMI reimbursement income. URRBMI reimbursement income = (medical expenditure – deductible) × reimbursement rate (here we assume that medical expenses have not exceeded the cap).

The redistributive effect is obtained by comparing the Gini coefficient computed on income after self-payment but before reimbursement with that computed on income after reimbursement. The resulting MT index and RMT quantify how the benefit payment compresses (or expands) the income distribution among the insured.

Illustrative example of income changes under URRBMI redistribution. Consider a middle-income individual whose initial disposable income equals the local annual per-capita amount *W*_*r*_. Region-specific URRBMI parameters are: deductible 0.02*W*_*r*_, coinsurance 78%, flat premium 0.01*W*_*r*_. According to Table 3, the probability that the middle-income group incurs a mid-tier inpatient episode is 0.2. Let medical expenses for this episode be 0.20*W*_*r*_. The four-step evolution of disposable income is then:

1. Pre-enrolment: initial disposable income = *W*_*r*_.
2. After premium: *W*_*r*_ − 0.01*W*_*r*_ = 0.99*W*_*r*_.
3. After out-of-pocket payment: 0.99*W*_*r*_ – 0.20*W*_*r*_×0.2 = 0.95*W*_*r*_.
4. After reimbursement: 0.95*W*_*r*_ + (0.20*W*_*r*_ – 0.02*W*_*r*_) × 0.78 = 0.95*W*_*r*_ + 0.14*W*_*r*_ ≈ 1.09*W*_*r*_.

**Table 3:** Changes in the Gini coefficient after inpatient spending under URRBMI (insured population; except for the Gini coefficient, all figures are expressed as multiples of *W*_*r*_).

**Income group (quintile)**		**1**	**2**	**3**	**4**	**5**	**Gini**
1. Initial disposable income		0.3	0.6	1	2	3	0.3942
2. After premium payment		0.29	0.59	0.99	1.99	2.99	0.3971
3. After out-of-pocket payment	By medical-expenditure bracket						
	0.02*W*_*r*_ (deductible)	0.288	0.587	0.986	1.985	2.984	0.3977
	0.08*W*_*r*_	0.278	0.576	0.974	1.972	2.97	0.4006
	0.14*W*_*r*_	0.262	0.562	0.962	1.962	2.962	0.4054
	0.2*W*_*r*_	0.24	0.545	0.95	1.955	2.96	0.4120
	0.26*W*_*r*_	0.212	0.525	0.938	1.951	2.964	0.4206
4. After insurance reimbursement	By medical-expenditure bracket						
	0.02*W*_*r*_ (deductible)	0.288	0.587	0.986	1.985	2.984	0.3977
	0.08*W*_*r*_	0.3248	0.6228	1.0208	2.0188	3.0168	0.3872
	0.14*W*_*r*_	0.3556	0.6556	1.0556	2.0556	3.0556	0.3789
	0.2*W*_*r*_	0.3804	0.6854	1.0904	2.0954	3.1004	0.3727
	0.26*W*_*r*_	0.3992	0.7122	1.1252	2.1382	3.1512	0.3683

Source: authors' calculations.

Applying the same algorithm to the five quintiles (0.3*W*_*r*_, 0.6*W*_*r*_, *W*_*r*_, 2*W*_*r*_, and 3*W*_*r*_) and to the five medical-expenditure brackets (0.02*W*_*r*_, 0.08*W*_*r*_, 0.14*W*_*r*_, 0.20*W*_*r*_, and 0.26*W*_*r*_) yields the income vectors reported in Table 3. The following points can be observed: (1) Premium payment. Because the contribution is a fixed absolute amount, the post-premium Gini rises slightly, indicating a mild disequalising effect. (2) Out-of-pocket stage. The Gini after self-payment exceeds the post-premium Gini and rises monotonically with the size of the medical bill, confirming that un-reimbursed hospital expenses widen the income gap. (3) Reimbursement stage. Except for the lowest expenditure bracket (equal to the deductible), the post-reimbursement Gini is lower than the post-payment Gini, signaling that the benefit payment produces a positive redistributive effect.

Table 4 presents the key indicators of the redistributive effect of URRBMI reimbursements. The following points can be observed: when expenditure equals the deductible (0.02*W*_*r*_), no reimbursement is triggered; hence redistribution is absent (MT = 0). For all expenditure levels above the deductible, G^*^ < G, confirming a positive redistributive effect. Both the MT index and the *RMT*_*m*_ rise monotonically with the size of the claim: larger hospital bills generate larger absolute and relative reductions in income inequality.

**Table 4:** Key indicators of URRBMI reimbursement redistribution (insured population).

**Inpatient-expenditure bracket**	**Gini_before (after out-of-pocket payment)**	**Gini_after (after reimbursement)**	**MT index**	**RMT_m_**
0.02*W*_*r*_ (deductible)	0.3977	0.3977	0	0.00%
0.08*W*_*r*_	0.4006	0.3872	0.0134	3.34%
0.14*W*_*r*_	0.4054	0.3789	0.0265	6.54%
0.2*W*_*r*_	0.4120	0.3727	0.0393	9.54%
0.26*W*_*r*_	0.4206	0.3683	0.0523	12.43%

Source: authors' calculations.

#### 3.2.2 Scenario 2 comparing redistribution between the insured and the uninsured

We now juxtapose the redistributive outcome of (i) enrolment plus reimbursement against (ii) remaining uninsured and paying the full hospital bill. The procedure is straightforward: compute the Gini coefficient of post-reimbursement incomes for the insured and compare it with the Gini coefficient that would prevail if the same population remained uninsured and bore the entire medical cost themselves. Table 5 shows the latter Gini values for each expenditure bracket. When the five income groups are not covered by URRBMI, they must pay the full inpatient bill out-of-pocket. Consequently, the Gini coefficient rises monotonically with the size of the bill: the larger the un-reimbursed expense, the greater the inequality among the quintiles.

**Table 5:** Changes in the Gini coefficient after inpatient spending (uninsured population; Except for the Gini coefficient, all figures are expressed as multiples of *W*_*r*_).

**Income quintile**		**1**	**2**	**3**	**4**	**5**	**Gini**
Initial disposable income		0.3	0.6	1	2	3	0.3942
Income net of inpatient expenditures	By inpatient-expenditure bracket						
	0.02*W*_*r*_ (deductible)	0.298	0.597	0.996	1.995	2.994	0.3948
	0.08*W*_*r*_	0.288	0.586	0.984	1.982	2.98	0.3977
	0.14*W*_*r*_	0.272	0.572	0.972	1.972	2.972	0.4024
	0.2*W*_*r*_	0.25	0.555	0.96	1.965	2.97	0.4090
	0.26*W*_*r*_	0.222	0.535	0.948	1.961	2.974	0.4175

Source: authors' calculations.

Next, we compare the Gini coefficient obtained when the entire population remains uninsured and pays the full inpatient bill (from Table 5) with the Gini coefficient observed after URRBMI reimbursement (from Table 4). The results are reported in Table 6. At the deductible (0.02*W*_*r*_) no reimbursement occurs; the MT index is negative, implying that remaining uninsured yields the marginally better distributional outcome. For every expenditure level above the deductible, the post-reimbursement Gini is lower than the uninsured Gini, and the MT index turns positive. Hence enrolment plus reimbursement unequivocally outperforms non-coverage. Both the absolute gap (MT index) and the relative advantage of being insured rise monotonically with the size of the hospital bill: the larger the expense, the greater the inequality-mitigating effect of URRBMI.

**Table 6:** Redistributive comparison: insured with reimbursement vs. uninsured.

**Inpatient-expenditure bracket**	**Gini (uninsured + full self-payment)**	**Gini (insured + reimbursed)**	**MT index**
0.02*W*_*r*_ (deductible)	0.3948	0.3977	−0.0029
0.08*W*_*r*_	0.3977	0.3872	0.0105
0.14*W*_*r*_	0.4024	0.3789	0.0235
0.2*W*_*r*_	0.4090	0.3727	0.0363
0.26*W*_*r*_	0.4175	0.3683	0.0492

Source: authors' calculations.

## 4 Empirical measurement

The empirical assessment of URRBMI redistribution relies on the 2019 China Household Finance Survey (CHFS). Conducted by the Survey and Research Center for China Household Finance at Southwestern University of Finance and Economics, CHFS is a nationally-representative panel survey that collects detailed micro-level information on household finances. Initiated in 2011, the survey is administered every 2 years; the 2019 wave (the fifth round) covers 34,643 households in 343 districts and counties across 29 provinces (autonomous regions and municipalities), yielding nationally and provincially representative data. CHFS 2019 contains rich modules on demographics, employment, assets and liabilities, income and consumption, social security and insurance, inter-generational transfers, and payment behavior. The variables most relevant to URRBMI are listed below.

f2001a records the type of social medical insurance held by the household member, with the following options: (1) Urban Employee Basic Medical Insurance, (2) Urban Resident Basic Medical Insurance, (3) New Rural Cooperative Medical Scheme (NRCMS), (4) Urban-Rural Resident Basic Medical Insurance, and (5) Government-funded Medical Care. Because by 2019 nearly all provinces in China had merged NRCMS with the Urban Resident Basic Medical Insurance (see the Appendix A for the relevant policy integration timelines), respondents who selected options 2, 3, or 4 are grouped under the Urban-Rural Resident Basic Medical Insurance in the analysis. f2004 indicates the household member's medical insurance premium paid last year. f2024 reports the household member's inpatient expenditures last year, which are classified into high, medium, and low terciles according to the reported amounts. f2028 covers annual outpatient and pharmacy-related expenses—including registration fees, laboratory tests, physical examinations, and pharmaceuticals—and is likewise divided into high, medium, and low terciles based on the reported values. f2025 captures the inpatient reimbursement received by a household member last year, broken down as follows: f2025_1: reimbursement under UEBMI. f2025_2: reimbursement under the Urban Resident Basic Medical Insurance. f2025_3: reimbursement under NRCMS. f2025_4: reimbursement under URRBMI. f2025_5: reimbursement under Government-funded Medical Care. We aggregate f2025_2, f2025_3 and f2025_4 into a single series labeled R2, representing the household member's total inpatient reimbursement under URRBMI. f2029 captures the non-inpatient reimbursement received last year, with components: f2029_1: reimbursement under UEBMI. f2029_2: reimbursement under the Urban Resident Basic Medical Insurance. f2029_3: reimbursement under NRCMS. f2029_4: reimbursement under URRBMI. f2029_5: reimbursement under Government-funded Medical Care. Likewise, we aggregate f2029_2, f2029_3 and f2029_4 into a series labeled Q2, representing the household member's total non-inpatient reimbursement under URRBMI. Table 7 summarizes these key URRBMI-related variables in the 2019 CHFS dataset.

**Table 7:** Key URRBMI-related variables in the 2019 CHFS database.

**Variable**	**Description**	**Treatment**
f2001a	Type of social medical insurance for each household member	Responses coded 2, 3, or 4 are grouped together and treated as URRBMI enrolment
f2004	Household's annual medical-insurance contribution	Used as reported
f2024	Household's annual inpatient expenditure	Recoded into high, medium, and low brackets
f2028	Household's annual non-inpatient medical expenses	Recoded into high, medium, and low brackets
f2025	Inpatient reimbursement received by the household	f2025_2, f2025_3, and f2025_4 are summed into one series labeled R2, representing total inpatient reimbursement under URRBMI
f2029	Non-inpatient reimbursement received by the household	f2029_2, f2029_3, and f2029_4 are summed into one series labeled Q2, representing total non-inpatient reimbursement under URRBMI

Source: 2019 CHFS database.

It should be noted that the CHFS database does not contain a ready-made variable for per-capita disposable income. We therefore construct it as follows: (1) household gross income is calculated as the sum of wage income + agricultural income + business income + property income + transfer income. (2) Household disposable income is obtained by subtracting from gross income: a3137 (individual income tax paid last year), f1008a (annual Urban-Rural Resident Basic Pension contributions), f1008 (monthly Employee Basic Pension contributions), f2004 (annual household medical-insurance contributions), and a flat RMB 230 per worker for unemployment-insurance contributions (based on the national standard of a 1% combined employer–employee rate^1^). (3) Per-capita disposable income is then derived by dividing the resulting household disposable income by a2000 (number of household members). In short, the per-capita disposable income used in our empirical analysis is already net of all social-insurance contributions, including the URRBMI premium.

### 4.1 National and regional estimates

Using inpatient expenditure as the illustration, Table 8 reports the redistributive impact of URRBMI for the full national sample and for the four major regions. The MT index is derived by comparing the Gini coefficient of disposable income after out-of-pocket hospital payments (post-premium income minus self-paid expenses) with the Gini coefficient after insurance reimbursement (post-OOP income plus URRBMI payout).

**Table 8:** Overall redistributive effect of urban-rural resident basic medical insurance (inpatient expenditures).

**Item**		**Gini after out-of-pocket payment**	**Gini after insurance reimbursement**	**MT index**
National		0.5891	0.5796	0.0096
By region	East	0.6106	0.5973	0.0133
	Central	0.5602	0.5600	0.0002
	West	0.5978	0.5831	0.0147
	Northeast	0.5127	0.4740	0.0386

Source: calculated by the authors using Stata and the 2019 CHFS database.

Based on the estimates presented in Table 8, across all specifications the MT index is positive, confirming that URRBMI narrows income inequality. Disaggregated by region, the redistributive effect is strongest in the Northeast, followed by the West and the East, while it is weakest in Central China. Nonetheless, the overall redistributive capacity of the resident medical insurance scheme remains weak across all regions.

### 4.2 Estimates by medical-expenditure brackets

We next estimate the redistributive effect of URRBMI across different expenditure levels. The procedure is as follows: (1) inpatient care. Variable f2024 (annual household inpatient expenditure) is divided into high-, medium- and low-cost brackets. For each bracket we calculate the Gini coefficient after out-of-pocket payment, and the Gini coefficient after URRBMI reimbursement. The MT index is then derived from the two Ginis. (2) Non-inpatient care. Variable f2028 (annual household non-inpatient medical expenses) is likewise split into high, medium and low brackets, and the same two-step Gini comparison is performed.

Table 9 reports the redistributive effects of URRBMI estimated with the national sample, stratified by medical-expenditure terciles. Three patterns emerge: (1) low-expenditure bracket: for low inpatient costs, the MT index is slightly negative (−0.0015); for low non-inpatient costs it is zero. Thus, URRBMI produces little or no redistribution at the lowest spending levels. (2) Medium- and high-expenditure brackets: both inpatient and non-inpatient reimbursements generate positive MT indices, indicating a progressive effect once expenditure exceeds the low-cost threshold. (3) Monotonic improvement: whether for inpatient or outpatient care, the MT index rises monotonically with the expenditure bracket. For inpatient reimbursement, for example, the MT index climbs from −0.0015 (low) to 0.0578 (high), mirroring the theoretical model's prediction that larger claims yield stronger redistribution.

**Table 9:** URRBMI redistributive effect by medical-expenditure bracket (national sample).

**Expenditure bracket**	**Inpatient reimbursement**			**Outpatient/non-inpatient reimbursement**		
	**Gini after OOP**	**Gini after reimbursement**	**MT index**	**Gini after OOP**	**Gini after reimbursement**	**MT index**
Low	0.5261	0.5276	−0.0015	0.5177	0.5177	0
Medium	0.5892	0.5774	0.0119	0.5359	0.5343	0.0094
High	0.7159	0.6581	0.0578	0.6024	0.5971	0.0406

Source: authors' calculations using Stata and the CHFS database.

## 5 Outstanding problems

Drawing on the foregoing analysis and the current operation of the scheme, the author identifies the following weaknesses in the present URRBMI design.

### 5.1 The flat-rate contribution mechanism undermines sustainability

The existing flat premium is regressive and jeopardizes the long-term viability of URRBMI. (1) Regressive financing. Because every enrollee pays the same absolute amount, high-income households bear a lighter burden (in percentage-of-income terms) than low-income households. This creates an implicit transfer from the poor to the rich and weakens the redistributive intent of the scheme. (2) Rapid contribution escalation. As shown in Table 10, between 2018 and 2024 the individual premium rose from RMB 220 to RMB 400—an 81.82% increase. Over the same period, national per-capita disposable income grew by only 46.36% [from RMB 28,228 (37) to RMB 41,314 (38)]. The premium is therefore outpacing income growth, especially for low-income groups. (3) Erosion of coverage. According to the Statistical Bulletin on the Development of China's National Medical Security, the number of URRBMI enrollees fell from 1,027.8 million at end-2018 to 962.9 million at end-2023 (−6.31%). While some exits are attributable to migration to employee schemes or the elimination of duplicate registrations after the launch of the unified national platform, the rising premium is indisputably another major driver of attrition. (4) Moral-hazard and equity issues. The flat premium neither rewards continuous participation nor imposes higher obligations on those with greater capacity to pay. High-income households effectively free-ride on a system financed disproportionately by low-income contributors, eroding both equity and sustainability.

**Table 10:** Individual contribution levels for urban-rural resident basic medical insurance, 2018–2024.

**Year**	**2018**	**2019**	**2020**	**2021**	**2022**	**2023**	**2024**
Individual contributions (RMB)	220	250	280	320	350	380	400

Source: compiled from annual announcements on the official website of the Central People's Government of China.

### 5.2 Benefit disparities run counter to the shared-prosperity goal

Large gaps in protection between URRBMI and UEBMI generate de facto inequity. (1) Vastly lower benefits under URRBMI. NHSA data for 2023 show that UEBMI covers 84.6% of in-directory inpatient costs (83.5%, 87.4%, and 89.4% at tertiary, secondary and primary facilities), while URRBMI covers only 68.1% (63.2%, 72.4%, and 80.8%^2^). Moreover, the average inpatient bill is RMB 12,175 for UEBMI vs. RMB 7,674 for URRBMI. These disparities blunt the overall redistributive impact of basic insurance. (2) Resource misallocation and tiered reimbursement. The benefit schedule penalizes higher-level hospitals: in Beijing, URRBMI reimburses 80% at primary-care facilities, 78% at secondary hospitals, and 75%−78% at tertiary hospitals (32). Although higher-level institutions possess superior staff, equipment, and technology, they impose higher deductibles and lower reimbursement rates. By contrast, primary-care providers are more accessible, feature lower deductibles, and offer higher reimbursement ratios. Yet URRBMI has historically reimbursed only down to township-level clinics; village-level stations are often excluded, which effectively limits rural residents' real-world access to care. (3) Income inequality readily translates into health inequality under URRBMI. The scheme assumes equal illness probability across income groups, yet utilization rises with both health awareness and income. A flat premium therefore forces low-income, low-utilization households to cross-subsidize high-income, high-utilization patients who prefer tertiary hospitals—an adverse redistribution. Moreover, fixed premiums and deductibles ignore ability-to-pay; for minor illnesses it is rational to remain uninsured. Low-income individuals consequently drop out, postpone care and risk falling seriously ill without coverage, while high-income members maintain continuous insurance and readily clear the deductible. Over time, delayed treatment and selective exit raise severe-disease incidence and future fiscal costs, widening health disparities and eroding both health welfare and the shared-development principle.

### 5.3 Heavy reliance on fiscal subsidies and weak risk-pooling

URRBMI funds are overwhelmingly dependent on government transfers and exhibit limited cross-subsidization. Table 11 shows the composition of URRBMI revenues between 2018 and 2022. Premium income alone has never been sufficient to cover annual outlays; without fiscal subsidies the scheme would run persistent deficits. In 2022, for example, contributions totalled only RMB 357.38 billion—far below the RMB 935.27 billion spent—while a subsidy of RMB 635.57 billion was required to balance the books and generate a small surplus. Fiscal transfers have consistently accounted for 60%−70% of total fund inflows over the period. Unless the contribution model is reformed and the pooling level raised, the fiscal burden will escalate further as benefit generosity increases. The overall mutual-support capacity of China's basic medical insurance fund system remains weak. While URRBMI is fiscally stretched, the employee scheme (UEBMI) posted a 2023 surplus of RMB 492.75 billion in its social-pooling account and accumulated reserves of RMB 2.63 trillion; in the same year, the employee medical insurance individual accounts recorded a current-period surplus of RMB 25.339 billion and an accumulated balance of 1,395.42 billion yuan.^3^ When these two components are combined, the basic medical insurance fund's accumulated balance exceeds RMB 4 trillion—enough to cover roughly 20 months of payments.^4^ But these funds cannot be reallocated to the lower-contribution URRBMI population. The resulting annual erosion of value runs into tens of billions of yuan, severely constraining the redistributive capacity of URRBMI.

**Table 11:** URRBMI fund revenues and expenditures, 2018–2022 (all figures in RMB 100 million).

**Year**	**2018**	**2019**	**2020**	**2021**	**2022**
Total URRBMI revenue	7,967.64	8,679.84	9,193.93	9,905.41	10,170.39
Of which: premium contributions	2,487.07	2,773.31	3,048.69	3,244.65	3,573.77
Contribution share of total revenue	31.21%	31.95%	33.16%	32.76%	35.14%
Of which: fiscal subsidies	5,374.1	5,796.24	5,996.09	6,268.44	6,355.73
Subsidy share of total revenue	67.45%	66.78%	65.22%	63.28%	62.49%
Total URRBMI expenditure	7,269.45	8,271.05	8,271.69	9,328.99	9,352.68

Source: China Fiscal Yearbook, 2019–2023 editions.

## 6 Policy recommendations

Drawing on the foregoing analysis, the following measures are proposed to strengthen the redistributive impact of URRBMI.

### 6.1 Introduce mandatory enrolment and income-proportional contributions

(1) Make URRBMI participation compulsory. Overall, URRBMI has played a positive role in narrowing income disparities. Raising coverage is therefore the first step to amplifying its redistributive effect. Voluntary enrolment—appropriate during the early integration phase—no longer suffices: coverage is now shrinking (see the 2023 Statistical Bulletin). Legislation should therefore mandate participation, supported by refined subsidy rules, to lock in the social-insurance pool and bolster redistribution. (2) Replace the flat premium with an income-proportional contribution. Linking the premium to household disposable income would embed equity into the financing structure. A proportional rate lightens the load on low-income households while obliging high-income households to pay more, eliminating the current regressive transfer from poor to rich and ensuring that contribution capacity, rather than head-count, determines payment.

### 6.2 Narrow the benefit gap through multiple levers

(1) Rationalize deductibles and ceilings. Currently the deductible is set per episode. If a single bill falls below the threshold, the patient pays the full cost—an outcome that disproportionately burdens low-income households. We recommend replacing episode-based deductibles with an annual cumulative out-of-pocket threshold. This raises the effective reimbursement rate for inpatient care and discourages unnecessary admissions aimed merely at crossing the deductible. (2) Lower deductibles and raise reimbursement rates at secondary and tertiary hospitals. As the economy grows, payment ceilings should be indexed upward and unused annual ceilings should be rolled over to cushion catastrophic episodes. (3) Introduce tiered outpatient benefits. Waive the outpatient deductible at primary-care facilities. Apply progressively higher deductibles for outpatient visits to secondary and tertiary hospitals. This steers demand toward cost-effective settings and lightens the load on poorer patients. (4) Expand the primary-care network. Accredit at least one village clinic per administrative village as an URRBMI-designated provider and raise both the reimbursement rate and the outpatient ceiling for these facilities. (5) Set a timetable for convergence. A phased timetable should be established to progressively narrow the benefit gap between the employees' scheme and the residents' scheme, with the goal of merging the two into a single, universal programme and achieving full population coverage by the Fifteenth Five-Year Plan period (2026–2030).

### 6.3 Leveraging the pooling function of the medical-insurance fund to enhance risk-sharing

We recommend elevating the risk-pooling level across both the Urban-Rural Resident Basic Medical Insurance (URRBMI) and the Urban Employees' Basic Medical Insurance (UEBMI). As noted earlier, URRBMI faces pronounced financing pressures and relies heavily on fiscal subsidies. Raising its pooling tier within its own risk pool merely reallocates insufficient reserves across provinces, yielding only marginal improvements. According to 2023 data, although URRBMI enrollees account for 72.19% of all basic medical-insurance participants, the scheme's revenue constitutes only 31.55% of total basic medical-insurance revenue, its expenditure 37.07% of total expenditure, and its accumulated surplus accounts for a mere 15.98% of the total accumulated balance of China's basic medical-insurance funds. By contrast, UEBMI, covering 370 million employees, holds 84.02% of the system's accumulated surplus, while URRBMI, covering 960 million residents, holds only 15.98% (39). The substantial idle balances in UEBMI not only erode the fund's value through inflation but also constrain the overall redistributive capacity of the basic medical-insurance system. The most effective solution is therefore to integrate the current dual-track structure—UEBMI and URRBMI—into a single, universal, statutory scheme. All individual accounts under UEBMI should be abolished; contributions should flow entirely into the pooled fund and be shared by all participants. A national-level equalization reserve should be established to address regional imbalances, ensuring that residents in lower-income areas receive medical benefits on a more equitable basis.

It should be noted that the “grand merger” is not a technical tweak but a political-economy upheaval. First, high-contributing UEBMI enrolees would treat pooling as expropriation and fear benefit dilution, while URRBMI members face premium hikes and higher deductibles, fuelling inter-group hostility and nationwide media spats. Second, the operational cliff is steep: unified contribution rates, benefit baskets and reimbursement rules must be rewritten; two IT systems, two bureaucratic silos and two collection channels have to be integrated, implying colossal transition costs and multi-agency turf wars. Third, local governments regard UEBMI surpluses as “their own purse”; any central reallocation or cross-provincial adjustment will meet fierce administrative resistance, amplified by unions, enterprise federations and public hospitals anxious about revenue uncertainty. Fourth, the entrenched norm of “more pay, more gain” frames any egalitarian reform as a breach of contract, eroding trust and potentially sparking an exit wave. A gradualist route—phased contribution convergence, grandfathered benefits, transparent compensation and protracted political bargaining—is indispensable to turn the merger from an academic aspiration into a viable policy goal.

.

## Data Availability

The data analyzed in this study is subject to the following licenses/restrictions: access to the dataset requires prospective users to apply for authorization directly from the China Household Finance Survey and Research Center website. Requests to access these datasets should be directed to China Household Finance Survey and Research Center, https://chfs.swufe.edu.cn/.

## Footnotes

^1^Pursuant to the Notice on the Phased Reduction of Unemployment and Work-Related Injury Insurance Contribution Rates (Ministry of Human Resources and Social Security [2023] No. 19), the unemployment-insurance rate is maintained at 1% (combined employer and employee contributions). Work-related injury insurance is paid solely by employers; employees make no personal payment. In accordance with the Guiding Opinions on the Full Integration of Maternity Insurance and Employees' Basic Medical Insurance (General Office of the State Council [2019] No. 10), the maternity-insurance fund has been merged into the employees' basic medical-insurance fund and is collected under a unified levy. Employers pay the combined medical (including maternity) insurance premium; individuals do not contribute to the maternity-insurance portion.

^2^Data source: National Healthcare Security Administration. Statistical bulletin on the development of national medical security in 2023 (2024-07-25; https://www.nhsa.gov.cn/art/2024/7/25/art_7_13340.html).

^3^Data derived from the 2023 Statistical Bulletin on the Development of National Medical Security.

^4^Estimated based on data from the 2023 Statistical Communiqué on the Development of Human Resources and Social Security.

## Appendix

**Appendix A1:** Integration timetable of the urban–rural resident basic medical insurance by province.

**Year**	**Province (Municipality/Autonomous Region)**
2010	Tianjin, Chongqing
2012	Guangdong, Ningxia
2014	Qinghai, Shandong
2016	Shanghai, Zhejiang, Hebei, Hubei, Inner Mongolia, Jiangxi, Xinjiang, Hunan, Guangxi
2018	Heilongjiang, Henan, Shanxi, Jiangsu, Fujian, Sichuan, Yunnan, Gansu, Jilin, Anhui, Hainan, Guizhou, Shaanxi, Beijing, Tibet
2019	Liaoning

Source: compiled by the authors from the official websites of provincial human resources and social security departments and official media reports.

## References

[1] Eurostat. Sickness and Healthcare Expenditure Down in 2022. Brussels, Belgium: European Commission (2023). Available online at: https://ec.europa.eu/eurostat/web/products-eurostat-news/w/ddn-20231127-1 (Accessed June 1, 2025).

[2] OECD. Health at a Glance: Europe 2022: State of health in the EU Cycle. Paris, France: Organisation for Economic Co-operation and Development (2022). Available online at: https://www.oecd-ilibrary.org/social-issues-migration-health/health-at-a-glance-europe-2022_507433b0-en;jsessionid=oEsuq4xgfTN86UPU8khhprbQLj7OHgdFS6o5oZzW.ip-10-240-5-112 (Accessed June 20, 2025).

[3] ISSA. Recent Health Reforms in Europe. Geneva: ISSA Publishing (2024). Available online at: https://www.issa.int/analysis/recent-health-reforms-europe (Accessed June 20, 2025).

[4] Tracking Universal Health Coverage: 2023 Global Monitoring Report. Geneva, Switzerland: WHO & International Bank for Reconstruction and Development/World Bank (2023). CC BY-NC-SA 3.0 IGO. Available online at: https://iris.who.int/server/api/core/bitstreams/3d4572d2-30a5-4cf0-bf73-0062d677bbf0/content (Accessed 30 October, 2024).

[5] OECD. Social Protection System Review: A Toolkit, OECD Development Policy Tools. Paris, France: OECD Publishing (2018). Available online at: 10.1787/9789264310070-en (Accessed 02 March, 2025).

[6] ILO. World social protection report 2020-22: Regional companion report for Central and Eastern Europe and Central Asia. Geneva, Switzerland: International Labour Organization (2021). Available online at: https://www.ilo.org/wcmsp5/groups/public/@europe/@ro-geneva/@sro-budapest/documents/publication/wcms_831024.pdf (Accessed March 20, 2025).

[7] European Observatory on Health Systems and Policies. Finland: Health System Summary. Copenhagen, Denmark: World Health Organization Regional Office for Europe (2023). Available online at: https://iris.who.int/bitstream/handle/10665/366710/9789289059398-eng.pdf?sequence=1 (Accessed March 10, 2025).

[8] Ministry of Social Affairs and Health. Wellbeing Services Counties will be Responsible for Organising Health, Social and Rescue Services. Helsinki, Finland: Ministry of Social Affairs and Health of Finland (2023). Available online at: https://stm.fi/en/wellbeing-services-counties (Accessed March 20, 2025).

[9] Kangas O Kalliomaa-Puha L. Finland Finalises its Largest-Ever Social and Healthcare Reform. Brussels, Belgium: European Commission (2022). Available online at: https://ec.europa.eu/social/BlobServlet?docId=25947&langId=en (Accessed March 10, 2025).

[10] ISSA. Reforms in Africa to Achieve Universal Health Coverage. Geneva, Switzerland: ISSA Publishing (2023). Available online at: https://www.issa.int/analysis/reforms-africa-achieve-universal-health-coverage (Accessed 30 October, 2024).

[11] ISSA. Improving Health Insurance Systems, Coverage and Service Quality. Geneva, Switzerland: ISSA Publishing (2021). Available online at: https://www.issa.int/analysis/improving-health-insurance-systems-coverage-and-service-quality (Accessed 30 October, 2024).

[12] Klein TJ Salm M Upadhyay S. Patient Cost-Sharing and Redistribution in Health Insurance, HEDG Working Paper, University of York (2024). Available online at: http://www.york.ac.uk/economics/postgrad/herc/hedg/wps/ (Accessed 02 March, 2025).

[13] Razavi S Cattaneo U Schwarzer H Visentin A. Combating Inequalities: What Role for Universal Social Protection? ILO Working Paper No. 128. Geneva, Switzerland: International Labour Office (2024) (Accessed 02 March, 2025) 10.54394/EOAY4970

[14] National Bureau of Statistics of China. China Data. Available online at: https://data.stats.gov.cn/easyquery.htm?cn=C01 (Accessed 11 December, 2024).

[15] National Healthcare Security Administration. Statistical Bulletin on the Development of the National Medical Security Service 2023 (2024). Available online at: https://www.nhsa.gov.cn/art/2024/7/25/art_7_13340.html (Accessed December 11, 2024).

[16] Pu X Wang Y Zhang W Zeng M. Can basic medical insurance reduce elderly family income inequality in China? Front Public Health. (2022) 10:838733. 10.3389/fpubh.2022.838733 35242735 PMC8885622

[17] Qin C Wang X. Does the increase in health insurance benefits have different effects on the health of middle-aged and older individuals in rural areas? Analysis based on quantile difference-in-differences method. Front Public Health. (2024) 12:1322790. 10.3389/fpubh.2024.1322790 38686030 PMC11056591

[18] Chen C Feng Z Gu J. Health, health insurance, and inequality. Int Econ Rev. (2024) 66:107–41. 10.1111/iere.12722

[19] Kaneva M Gerry CJ Avxentiev N Baidin V. Attitudes to reform: could a cooperative health insurance scheme work in Russia? Int J Health Econom Manage. (2019) 19:371–94. 10.1007/s10754-019-09260-3 30671697

[20] Bryndová L Hrobon P Tulejová H. The 2018 risk-adjustment reform in the Czech Republic: introducing pharmacy-based cost groups and strengthening reinsurance. Health Policy. (2019) 123:687–93. 10.1016/j.healthpol.2019.05.017 31196570

[21] Mulenga A Ataguba JE. Assessing income redistributive effect of health financing in Zambia. Soc Sci Med. (2017) 189:1–10. 10.1016/j.socscimed.2017.07.017 28755543

[22] Liao Z Yu J. Income redistribution effects of China's basic medical insurance system—empirical analysis based on China household finance survey data. Res Financ Econ Issues. (2021) 452:57–65. 10.19654/j.cnki.cjwtyj.2021.07.006

[23] Yang S Zhao X. Toward common prosperity: practice, effects, and insights of China's social security redistribution. Manag World. (2022) 38:43–56.

[24] Zhou Q Zhang Q Cai Z. Impact of rural medical insurance on income inequality among residents. J Zhongnan Univ Econ Law. (2021) 247:105–18. 10.19639/j.cnki.issn1003-5230.2021.0046

[25] Gu X Hui W. Redistribution Effects of Universal Health Security Under the Vision of Common Prosperity. Research on Financial and Economic Issues (in Chinese) (2022).

[26] Jin S Yu J Tian R. Does China's basic medical insurance promote benefit equity? Evidence from the China Household Finance Survey. China Econ Q. (2020) 19:1291–314. 10.13821/j.cnki.ceq.2020.03.07

[27] State Council of the People's Republic of China. Guiding Opinions on Launching the Pilot Program of Basic Medical Insurance for Urban Residents (2007). Available online at: https://www.gov.cn/gongbao/content/2007/content_719882.htm (Accessed December 11, 2024).

[28] National Healthcare Security Administration Ministry Ministry of Finance State Taxation Administration. Notice on Properly Conducting Basic Medical Security for Urban and Rural Residents in 2024 (2024). Available online at: https://www.nhsa.gov.cn/art/2024/8/26/art_104_13632.html (Accessed December 12, 2024).

[29] Ministry of Health Ministry of Civil Affairs Ministry of Finance Ministry of Agriculture State State Administration of Traditional Chinese Medicine. Opinions on Consolidating and Developing the New Rural Cooperative Medical System (2009). Available online at: https://www.gov.cn/gongbao/content/2010/content_1555968.htm (Accessed December 11, 2024).

[30] State Council. Notice on Issuing the Immediate Key Implementation Plan (2009–2011) for Healthcare Reform. State Council Gazette (2009). Available online at: https://www.gov.cn/gongbao/content/2009/content_1284376.htm (Accessed December 11, 2024).

[31] Ministry of Human Resources and Social Security Ministry of Finance. Circular on Properly Conducting Basic Medical Insurance for Urban Residents in 2010 (2010). Available online at: https://www.mohrss.gov.cn/SYrlzyhshbzb/shehuibaozhang/zcwj/yiliao/201006/t20100601_86864.html (Accessed December 11, 2024).

[32] Beijing Municipal Government. What are the Outpatient and Inpatient Reimbursement rates? Check here! (2024). Available online at: https://www.beijing.gov.cn/fuwu/bmfw/sy/jrts/202403/t20240306_3581600.html (Accessed December 11, 2024).

[33] National Bureau of Statistics. Statistical Communiqué of the People's Republic of China on the 2023 National Economic and Social Development (2024). Available online at: https://www.stats.gov.cn/sj/zxfb/202402/t20240228_1947915.html (Accessed December 11, 2024).

[34] OECD. Health for Everyone? Social Inequalities in Health and Health Systems, OECD Health Policy Studies. Paris, France: OECD Publishing (2019) (Accessed March 2, 2025). 10.1787/3c8385d0-en

[35] Musgrave RA Thin T. Income tax progression, 1929–1948. J Polit Econ. (1948) 56:498–514. 10.1086/256742

[36] Duan M Lou F. Measurement of redistributive effects of China's government regulatory tools. Inquiry Econ Issues (2023) 42:1–17. https://kns.cnki.net/kcms2/article/abstract?v=X-VFCYicIZvjptIlJqv0BoYNbci6c3acgG 92KyLNpA3FcyipEF0IHw0XhGZsBzDceiZcfnAu0YkFncSH3b1CPDHw6QKKcWGW 7NxDY_wVe2bdpMPqqHZF0Cnn13-XmnZ_nkbfaRZWQpUse3XXGDIe_JC-BTZm- 1uS2DLIJtaESph48MgVUq0aFA==&uniplatform=NZKPT&language=CHS

[37] National Bureau of Statistics. Income and Consumption Expenditure of Residents in 2018 (2019). Available online at: https://www.stats.gov.cn/sj/zxfb/202302/t20230203_1900203.html (Accessed April 15, 2024).

[38] National Bureau of Statistics. Income and Consumption Expenditure of Residents in 2024 (2025). Available online at: https://www.stats.gov.cn/sj/zxfb/202501/t20250117_1958325.html (Accessed April 15, 2024).

[39] National Bureau of Statistics. China Data (2025). Available online at: https://data.stats.gov.cn/easyquery.htm?cn=C01 (Accessed March 9, 2025).

